# Infectious scleritis: a review of etiologies, clinical features, and management strategies

**DOI:** 10.3389/fopht.2025.1493831

**Published:** 2025-02-07

**Authors:** Supriya Sharma, Jay U. Sheth, Somasheila I. Murthy

**Affiliations:** ^1^ Department of Cornea and Anterior Segment Services, Shantilal Shanghvi Eye Institute, Mumbai, Maharashtra, India; ^2^ Department of Vitreo-retina Services, Shantilal Shanghvi Eye Institute, Mumbai, Maharashtra, India; ^3^ Shantilal Shanghvi Cornea Institute, LV Prasad Eye Institute, Hyderabad, Telangana, India

**Keywords:** infectious scleritis, scleral debridement, pseudomonas scleritis, fungal scleritis, management of infectious scleritis, microbiological profile of infectious scleritis

## Abstract

Infectious scleritis is a severe and potentially vision-threatening inflammation of the sclera caused by microbial invasion. Unlike autoimmune scleritis, infectious scleritis is less common with a prevalence of 5%–10% of all cases of scleritis. The initial clinical presentation may often resemble autoimmune scleritis, thus resulting in a delayed diagnosis and poorer outcomes. A high level of suspicion is required in such cases and risk factors such as with a history of trauma or surgery and worsening or refractory to immunosuppressive medications as these may well be infectious. While in established cases, the classical clinical features enable us to diagnose as infectious etiology without much difficulty, the management remains a challenge. Majority of these cases require an aggressive approach with a combination of antimicrobial therapy and surgical treatment. Poor prognostic factors include delayed diagnosis, fungal etiology, and the presence of keratitis or endophthalmitis. In this review, the authors have summarized the prevalence, clinical and microbiological profile, and management strategies of infectious scleritis and their outcomes.

## 1 Introduction

Scleritis is a painful state of ocular inflammation with a wide spectrum of clinical presentation and etiological factors (1, 2). It is most often associated with autoimmune or connective tissue disease but may be idiopathic or have an infectious etiology, each of which presage varying severity and prognosis of the disease (3). Infection is an undesirable and rare cause of scleritis, involving deep inflammation of the scleral tissue, posing a significant threat to the globe integrity and vision.

Clinical differentiation between infectious scleritis (IS) and autoimmune scleritis is challenging during the early stages, thereby delaying the correct diagnosis and delaying the institution of appropriate medical therapy (4). Majority of these eyes are treated with immunosuppressive therapy alone, resulting in exacerbation of the infectious process and poorer final outcomes. However, a favorable prognosis can be obtained when early diagnosis and appropriate therapy is implemented. This underscores the critical importance of distinguishing between infectious and autoimmune causes early on (5).

## 2 Epidemiology

The prevalence of IS is largely determined by the geographical location. Infectious scleritis is a rare clinical entity and accounts for just 5%–10% of all cases of scleritis, with bacterial infection being the most common etiology (53%–87%) (3, 6). Although various organisms have been identified as the cause for IS, *Pseudomonas aeruginosa* remains the most common pathogen. Yu et al. reported this microbe to be the commonest etiological agent in about 87% of cases, whether reported from the United States, South Korea, Australia, or Singapore (3, 6–8). It is to be noted that fungal infection is far less common, seen in only about 11% of the cases in western cohorts (5). IS was also noted to be predominant in the middle-aged to elderly patients (mean age ranging from 40 to 70 years), with a female preponderance (57%–70%). Infectious scleritis represents a higher percentage of scleritis cases in developing countries, such as India where it accounts for 18%–30% (9, 10). Unlike the western literature, fungus has been reported to be the most common cause of IS in the Indian subcontinent (23.5 to 38%), followed by *Pseudomonas* spp. (range: 13.7%–24%) (3, 11). In the developing regions, IS most commonly affects younger individuals (mean age ranging from 40 to 50 years). Here men (49%–81%) were more commonly affected than women. Jain et al. have attributed the higher incidence of fungal infection in India to agricultural and environmental factors (3). *Aspergillus flavus* is the most common fungal isolate in the eyes with fungal scleritis. Moreover, systemic diseases such as tuberculosis is endemic to the Indian subcontinent, so it is not surprising to note that a few publications have reported that almost 75% cases of infectious scleritis are tuberculosis-related scleritis (3, 5).

## 3 Classification

**Figure 1:**
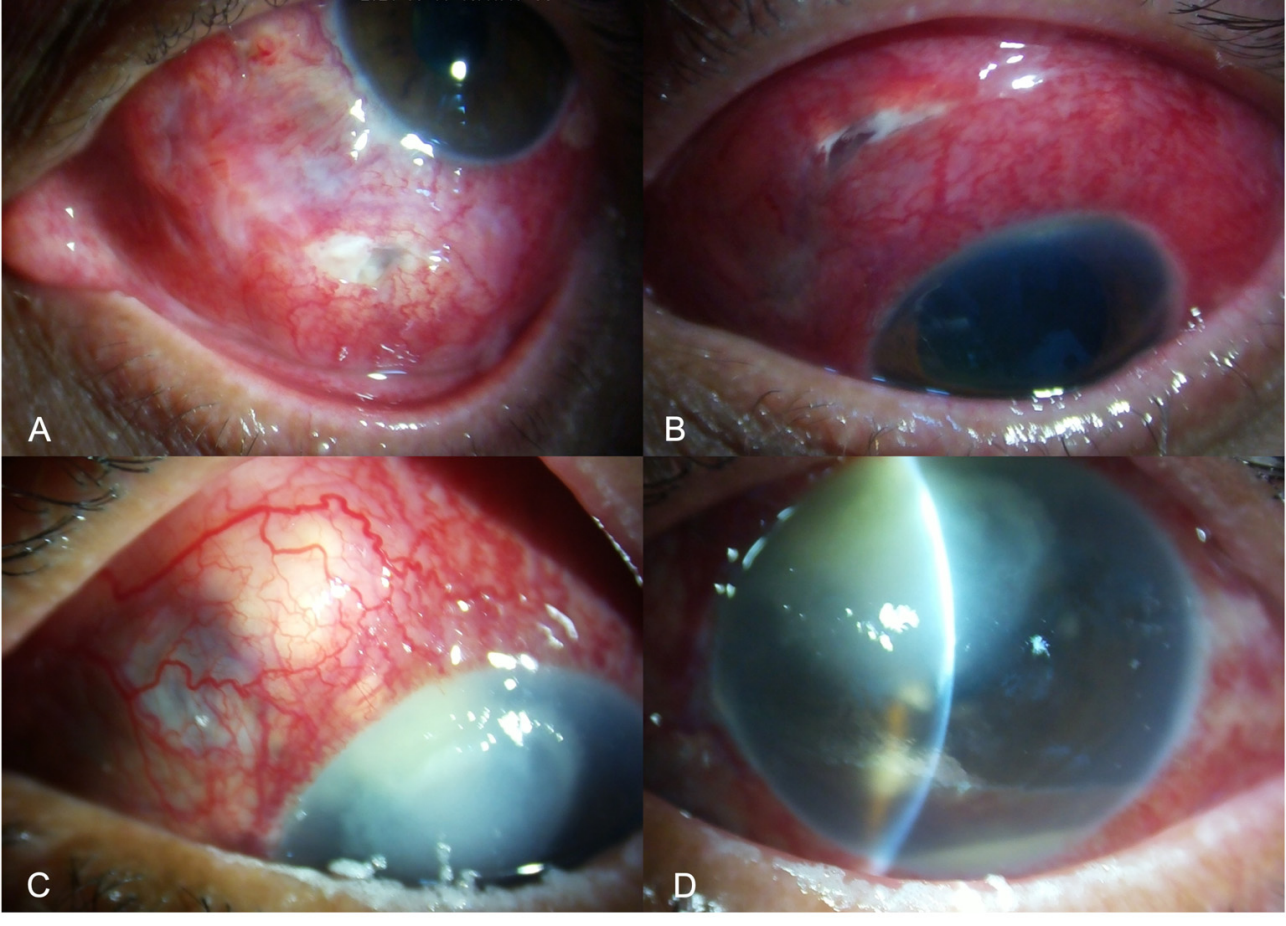
Slit-lamp image of the left eye of a patient presenting with infectious scleritis, a week after penetrating trauma with a wooden stick. The wound involves the inferior scleral tissue with infiltrate and conjunctival necrosis along the wound **(A)**. Slit-lamp image of the left eye in a patient with scleral infection and thinning involving the scleral tunnel following small incision cataract surgery. The nasal edge of the incision shows retraction of the conjunctival tissue with chemosis and infiltrate **(B)**. Slit-lamp image a patient with infective scleral nodule in the super-nasal quadrant **(C)**, due to spread of infection from the adjacent corneal infection following accidental injury with a twig **(D)**. Microbiological examination of the samples collected from the scleral nodule and corneal scraping showed fungal filaments.

All microbial agents such as bacteria, fungi, viruses, and parasites are reported to cause infectious scleritis, with coinfections caused by two or more clinical pathogens being far less common (4). The infection may have an exogenous or endogenous source. The exogenous route is far more common—due to external ocular wounds following trauma or surgery or spread from an adjacent ocular infection, such as microbial keratitis (**Figure 1**). These infections tend to be more acute and suppurative, presenting with a greater severity of tissue damage. Endogenous IS is an uncommon clinical entity and often resembles or is misdiagnosed as noninfectious diffuse, nodular, or necrotizing scleritis. Scleritis secondary to systemic infections such as tuberculosis, herpes virus, and syphilis also falls under this category.

IS may also be classified as a primary or secondary infection (4). Primary IS is a term reserved for those eyes where infection is seen as a direct consequence of scleral surgery or accidental ocular trauma. Predisposing factors to a primary infection include systemic infection, systemic/topical steroid use (4), and a previous diagnosis of autoimmune scleritis. Secondary IS is a term reserved for cases where the infection spreads from an adjacent infectious foci like keratitis or endophthalmitis. The risk factors for secondary IS are like those of primary IS, except for history of contact lenses use, presence of debilitating systemic diseases, and corneal tissue devitalization. Regardless of the classification, there are two pathological mechanisms contributing to the development of IS, i.e., direct microbial invasion causing tissue destruction or an immune response triggered by the infectious agent, as seen in eyes with tuberculous scleritis (5).

Posterior infectious scleritis is a rare but potentially destructive disease characterized by infection and inflammation of the posterior sclera. The emergence of posterior scleritis is accompanied by relatively few or, in some cases, no visible clinical signs at all, making diagnosis difficult. Retained intraocular foreign body (IOFB) has been previously reported to cause posterior infectious scleritis (4).

## 4 Risk factors

### 4.1 Surgery

**Figure 2:**
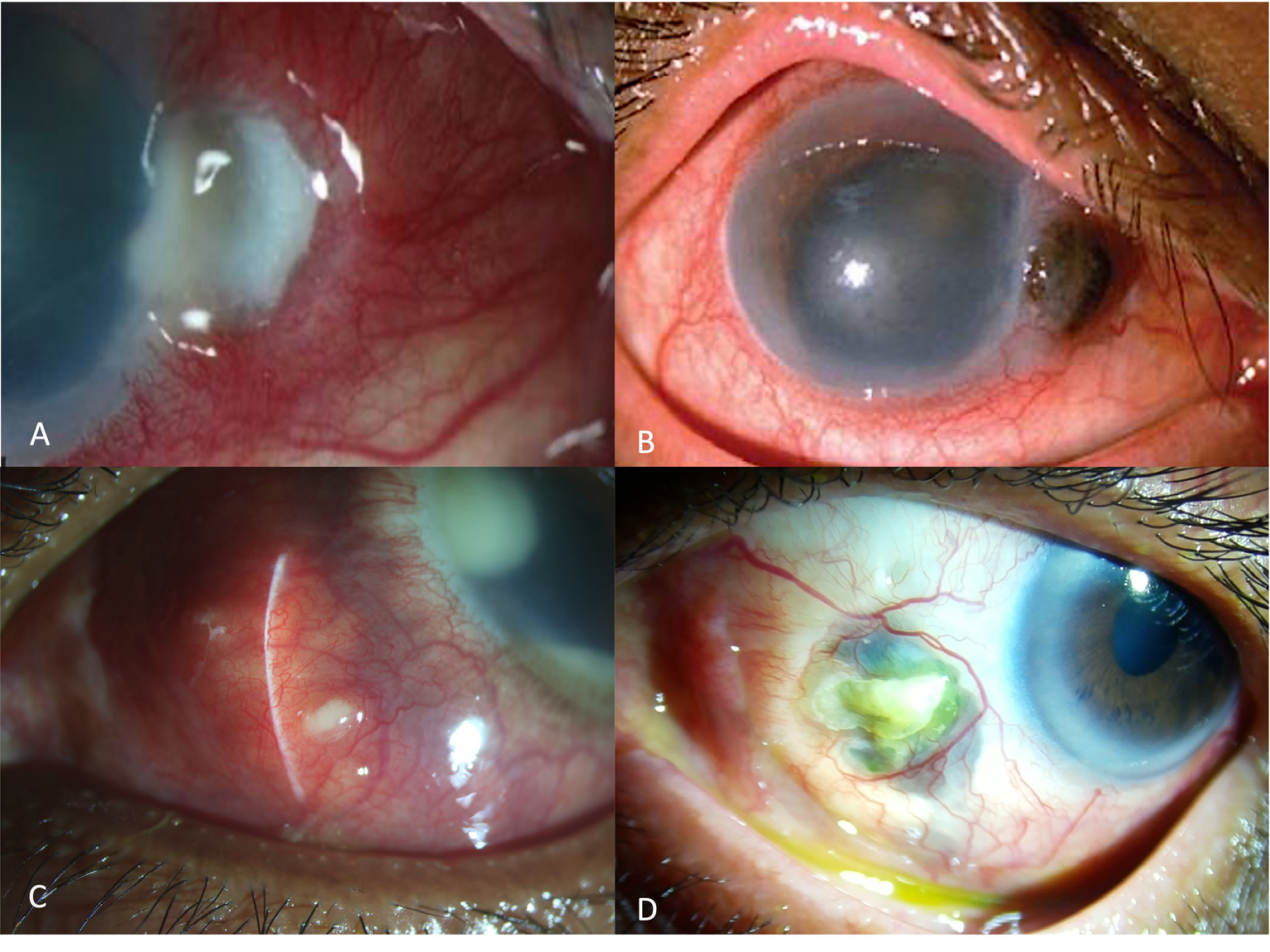
Slit-lamp image of the left eye showing scleral necrosis after pterygium excision, characterized by yellow infiltrate overlying the bare sclera with adjacent corneal stromal lysis **(A)**. Slit lamp image of the right eye of a patient presenting with corneo-scleral perforation after pterygium excision, due to longstanding infectious scleritis after pterygium surgery, recalcitrant to medical therapy **(B)**. Slit-lamp image of the right eye showing an infectious lesion, with a pus-point around the belt-buckle with contagious spread of infection involving the cornea, 3months after a vitreo-retinal surgery for retinal detachment **(C)**. Slit-lamp image of the left eye showing scleral necrosis, infiltrate with thinning and underlying uveal show around the area of the sclerotomy port following retinal surgery **(D)**.

Previous ocular surgery is a well-established risk factor for IS, with pterygium surgery being the most common surgical procedure (3, 5, 6). Excessive cauterization and bare sclera technique after pterygium excision increase the susceptibility of IS (**Figures 2A, B**). Incorrect surgical technique and surgery-induced trauma cause the destruction of the conjunctival and episcleral tissue, including their vascular supply, thus decreasing local tissue immune resistance and exposing the sclera to microbial pathogens. Tissue destruction is propagated by the production of collagenase and subsequent enzymatic degradation in response to the prolonged exposure of the bare sclera to the tear film and atmosphere. Additionally, the use of adjunctive beta irradiation or antimetabolites, such as mitomycin-C and thiotepa, prevents re-epithelization and leaves the sclera vulnerable to microbial invasion (12).

Jain et al. have reported cataract surgery to be the predisposing factor in 30% eyes, followed by pterygium surgery (24%) (3). The reported interval between the surgery and diagnosis of IS ranges from 1 week to 4 years following cataract surgery and 1 month to 3 years following pterygium surgery. The higher prevalence of IS following cataract surgery in developing countries may be attributed to the fact that a higher number of manual incision cataract surgeries are performed using a self-sealing but larger sclero-corneal tunnel compared to clear corneal phacoemulsification performed in the developed countries (13).

Other less common predisposing surgical procedures include vitreo-retinal surgeries such as pars plana vitrectomy (PPV) and scleral buckling (**Figures 2C, D**). The reported incidence in developing countries is around 6%–14% (3, 9). Reddy et al., in a retrospective study from a tertiary eye care center in India, reported that 75% of the cases with post-surgical IS had a history of vitreo-retinal surgery, especially scleral buckle surgery (10). Majority of these eyes developed infection related to the implanted scleral buckle and presented with severe chronic pain and decreased visual acuity at varying lengths of time in the postoperative period. Smiddy et al. reported that *Staphylococcus* species caused most of the culture-positive buckle infections. They reported that the organism formed a biofilm around the buckle, thus promoting bacterial sequestration and colonization and inhibiting the penetration of antibiotics (14). These cases are infrequently encountered in the recent decade as there has been a paradigm shift in retinal surgeries from scleral buckle to pars plana vitrectomy.

A newer and rare form of post-surgical infection was noted with the cosmetic surgeries like “eye-whitening” procedures, including regional conjunctivectomy with mitomycin C application, commonly performed in Southern Korea and I-BRITE procedure which is popular in the Unites States, with reports of scleral infection emerging in both. Less frequently, strabismus surgery and glaucoma filtration surgeries have also been known to cause IF (15).

### 4.2 Trauma

Direct implantation or seeding of the microorganisms in the scleral tissue following accidental penetrating trauma, especially with organic material, is a common cause of IS. Foreign bodies that lodge in or pass through the sclera may also harbor the organisms (5). *Nocardia* and *Aspergillus* are soil inhabitants that are associated with the majority of the post-traumatic IS cases (10, 16). Trauma to a distant site in the body may also cause IS through hematogenous spread or self-inoculation. Trauma and incorrect surgical technique cause destruction of the conjunctival and episcleral tissue and its vasculature, predisposing the sclera to be invaded by microorganisms. Additionally, the microbial invasion incites an inflammatory microangiopathy response in the vascular channels (4).

### 4.3 Iatrogenic

The use of topical and systemic steroids weakens the immune barrier predisposing the eye to IS (9, 17). Though the scleral tissue is radioresistant, IS has been seen in eyes with uveal melanoma that received radiotherapy. Sub-tenon triamcinolone acetonide injections have been known to cause early-onset (bacterial) and late-onset (fungal) infectious scleritis (18). Fungal infections by *Aspergillus* have been commonly associated with intravenous drug abuse (19).

### 4.4 Others

IS may present as a secondary spread of infection from adjacent structures, such as keratitis and endophthalmitis, through contiguous spread. Systemic immunosuppression is also a risk factor for IS. Diseases such as human immune-deficiency virus (HIV) infection and diabetes mellitus are commonly associated with IS (4, 20).

## 5 Clinical presentation

The clinical findings in the case of infectious scleritis, including the mode of onset, presentation, natural history, and the disease course, depend on the precipitating cause of the infection. Based on the above-mentioned features, Murthy et al. have elaborated the differentiating features between infectious and non-infectious scleritis (4).

### 5.1 Symptoms and signs

**Figure 3:**
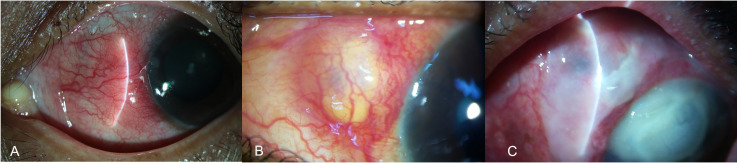
Slit-lamp images showing the various clinical features of infectious scleritis. In the early stages the patients may present with a subconjunctival nodule, with localized conjunctival congestion **(A)**. As the infection worsens the nodule increase in size and appears yellowish **(B)**. Eventually, as the infection spreads, scleral lysis with ulceration and necrosis of the overlying conjunctival is seen with the spread of infection to the adjacent structures like cornea and the anterior chamber **(C)**.

There are specific clinical symptoms that should raise a strong suspicion of scleritis, which include ocular pain, redness, watering, decrease in visual acuity headache, and photophobia. Clinical signs suggestive of infective etiology include scleral necrosis, calcified plaques, conjunctival ulceration and sloughing, hypopyon and anterior chamber reaction, corneal infiltrates, yellowish-white uni- or multifocal scleral abscesses or pus points, ulcerative scleral lesion, mucopurulent discharge, and scleral thinning (**Figure 3**) (10, 21). Exposure of the scleral buckle with surrounding necrosis and discharge, choroidal thickening, exudative retinal detachment, and widening of the sub-tenon’s space are suggestive of buckle-related IS.

Necrotizing scleritis is most frequently associated with IS (93%), with a yellowish or reddish hue of the lesion due to underlying suppuration (5). Multiple nodules may appear over the course of the disease, which may coalesce and lead to scleral necrosis and thinning. These nodules are mostly seen at around 4 to 5 mm from the limbus in a circumferential pattern. Eventually, a black arc-shaped band appears, extending from the initial lesion, across all the satellite lesions due to scleral thinning and uveal show. This is suggestive of intrascleral dissemination (5). Once these lesions heal, a fibrous band appears overlying the area of scleral thinning, unlike in eyes with immune-mediated scleritis, which heal only with scleral thinning. Poor visual acuity at presentation (<20/200) is associated with poor prognosis and worse visual outcomes, emphasizing the need for early diagnosis and treatment (13).

Patients with posterior infectious scleritis have been reported to present with blurred vision, deep-seated eye pain, particularly during extraocular movements that may radiate around the orbit, diplopia with reduced extraocular movements, and, in a few instances, proptosis, episcleral congestion chemosis, and lid edema. Choroidal mass, serous retinal detachments, usually at the posterior pole, retinal and/or disc edema, retinal and/or choroidal folds, choroidal detachment (CD), and vitritis are usually found on fundus examination. In a small number of cases, anterior uveitis may also be present (4, 22).

### 5.2 Mode of onset

The natural history, clinical course, and presenting features are indicative of the underlying cause in majority of the eyes with IS (4, 10). In eyes with exogenous IS, history of recent ocular surgery, trauma with organic matter, or presence of concurrent keratitis may help in diagnosing IS. Another characteristic feature of IS is delayed presentation, occurring months to several years after the ocular surgery or trauma (5). Latency is defined as the time interval between the inciting event and the onset of the symptoms. Although this time period is extremely variable, nonsurgical post-traumatic cases tend to have a shorter latency period (0–4 weeks) compared to post-surgical cases (1 week to 36 years). This can be explained by a greater ocular surface damage and a higher load of the infectious agent following trauma (4, 23, 24).

Delayed presentation is a characteristic feature of both IS and surgically induced necrotizing scleritis (SINS). Hence, post-surgical cases of IS should be differentiated from eyes with SINS, with the latter having a longer latency period (up to 36 years and up to 51 years, respectively) (25). The microorganisms may remain dormant inside the sclera tissue, without inciting an inflammatory response, making the diagnosis of IS extremely challenging (5). Secondary infection in eyes with SINS, in the absence of systemic vasculitis, has been reported previously. The underlying mechanism was possibly explained by *Lin* et al., where the authors postulated that after the onset of SINS, the microorganisms invade the scleral tissue, causing late-onset IS (26). Jain et al. proposed that tear film and conjunctival epithelial alterations may expose underlying necrotic scleral collagen to microorganisms and allow localization, adherence, colonization, and invasion (3).

### 5.3 The course of the disease

The course of IS is primarily determined by the virulence of the causative organism (7). Fastidious microorganisms like non-tubercular *Mycobacteria*, *Nocardia* sp., or fungi usually present as an indolent infection (3, 27). In contrast, microorganisms such as *Pseudomonas* sp. and *Staphylococcus* sp. or viral infections have a shorter and more aggressive clinical course (28, 29). Immune-mediated scleritis has a more protracted course determined by the underlying systemic disease (4). Once the systemic disease is in remission, the scleritis component also gradually subsides. The time to resolution after starting the treatment is known to be longer in eyes with IS compared to those with an immune-mediated disease.

### 5.4 Response to immunosuppressive treatment

Topical and systemic immunosuppressants reduce the inflammatory reaction, causing significant symptomatic relief (30, 31). Patients with IS may notice a marked reduction in pain and redness, particularly with topical steroids. This delays the diagnosis, causing the organism to proliferate. Therefore, in any patient with a scleral lesion that initially responds to immunosuppressants, especially topical steroids, but then worsens with subsequent features as described in this review, the clinician should be alerted to the possibility of an infectious cause (32).

### 5.5 Atypical clinical presentation

Atypical IS is characterized by painless presentation, unaffected visual acuity, lack of corneal involvement, absence of anterior chamber reaction, and normal intraocular pressure (5). IS may commonly masquerade as surgically induced necrotizing scleritis (SINS), ocular tumors, orbital cellulitis, carotico-cavernous fistula, cavernous sinus thrombosis, and autoimmune scleritis (6). Isolated case reports support atypical clinical presentations like aqueous fistula in pneumococcal scleritis, a case of pyomyositis presenting with an inverse hypopyon, and spontaneous IS in immunocompromised patients (33–35). Atypical IS presents with extreme heterogeneity, and a high level of clinical suspicion and thorough diagnostic evaluation before labeling any scleral lesion as IS.

## 6 Etiology and microbe-specific clinical features

### 6.1 Bacteria

Bacterial scleritis significantly contributes to cases of IS, particularly in developed countries (6, 23). These pathogens cause tissue destruction through proteases enzyme produced by the microbes and the activated neutrophils and activation of tissue complement pathways (36).

#### 6.1.1 Gram-negative bacteria

Bacteria are the most common cause of IS in developed countries. Among them, *Pseudomonas* sp., particularly *Pseudomonas aeruginosa*, is the most common causative organism contributing to almost 51%–85% cases of IS in the west, most often associated with necrotizing scleritis, corneal infection with subsequent scleral infection, and IS post-pterygium surgery (20, 26, 28). The infection is often severe and associated with poor prognosis due to excessive tissue destruction and collagenolysis caused by the enzymes produced by the activated inflammatory cells and the pathogen itself. *Stenotrophomonas maltophilia* is another less common pathogen known to cause IS following pterygium surgery and secondary to corneal infection (37, 38). The organism has an indolent course, is less fulminant, and is collagenase-negative, causing lesser tissue destruction compared to the previous pathogen. However, the challenge lies in the treatment as the organism is multi-drug resistant. Lin et al. reported a case of *S. maltophilia*-associated IS that occurred 18 years after a pterygium surgery. The authors also highlighted the importance of long-term follow-up and therapy after a case of IS that recurred and presented with intrascleral dissemination 5 months after complete resolution (38). Esteben and Jeng highlighted the importance of ultrasound biomicroscopy in a case of IS that revealed a dome-shaped mass overlying an area of partial-thickness scleral laceration after minor ocular trauma.

Other gram-negative organisms known to cause IS include *Serratia marcescens*, *Enterobacter cloacae*, *Escherichia strain*, *Proteus* sp., and others (6, 10, 12, 24).

#### 6.1.2 Gram-positive bacteria

*Staphylococcus aureus* is frequently associated with post-pterygium and vitreo-retinal surgeries. A staphylococcal infection may also spread from a distant site in the body (24, 39, 40). Arora et al. reported a case of *S. aureus* IS which presented as pyomyositis with inverse hypopyon and localized scleral thinning in a patient with a large thigh abscess (39). When the infection shows poor or no response to the typical b-lactam class of antibiotics, a possibility of multi-drug-resistant methicillin-resistant *S. aureus* (MRSA) scleritis should be considered (29). This was highlighted by Lee et al. who reported a case of MRSA 6 months after a pterygium surgery. An initial misdiagnosis of *Pseudomonas*-associated IS was reconsidered as the lesion showed no response to systemic amikacin and ceftazidime as well as topical ciprofloxacin (29).

*Streptococcus pneumoniae* infection represents a potentially aggressive form of the disease, commonly seen in children and adults with concomitant bone, joint, cardiovascular, gastrointestinal, or genitourinary infection. *S. pneumonia*-related IS is an aggressive form of disease requiring a combination of topical, intravenous, and oral medications (33, 41).

Other gram-positive organisms known to cause IS include Enterococcus faecalis, Propionibacterium acnes, Corynebacterium diphtheriae, Listeria monocytogenes, Actinomyces spp., and others (10, 24, 42).

#### 6.1.3 Nocardia scleritis

*Nocardia* spp. are gram-positive, weakly acid-fast organisms which morphologically resemble fungi due to their filamentous branching structures (3). A disease caused by *Nocardia* has a long course, with non-specific clinical signs and difficult microbiological analysis. It is important to consider nocardiosis in cases of unilateral anterior scleritis or sclerokeratitis, combined with a trauma history involving organic materials or a recent ophthalmic surgery (11). Diagnosis is often delayed due to unfamiliarity with the pathogen and poorly understood treatment alternatives, particularly in drug-resistant cases, resulting in potential sight-threatening complications. The commonly reported predisposing factors include cataract surgery, scleral buckling surgery, penetrating keratoplasty, sub-tenon triamcinolone acetonide injection for cystoid macular edema, trauma, and contact lens wear (11, 16).

*Nocardia* is a saprophytic bacterium genus found mostly in soil, water, dust, and decaying waste. India, being an agriculturally oriented emerging country, gives more opportunity for this organism to be transferred from the soil to the eye (3, 11). *Nocardia asteroids* is the most often reported organism, followed by *Nocardia nova*. The classical clinical presentation is chronic diffuse scleritis with nodules and/or necrotic abscesses. Studies from India have reported the incidence of *Nocardia* keratitis ranging from 14% to 25% (3, 10, 11, 13). Sahu et al. reported three cases of *Nocardia* scleritis and published an extensive review of the published literature highlighting the importance of surgical debridement in addition to topical and systemic medications to achieve complete resolution (11).

### 6.2 Fungal scleritis

The reported incidence of fungal keratitis in developing and tropical countries ranges from 5% to 38% (3, 10, 24). The high prevalence of fungal keratitis has been attributed to geographical locations having a hot and humid climate and the abundance of fungal spores in the environment. The infection can spread endogenously in immunocompromised people or through intravenous drug use, or it can also be transmitted exogenously by trauma or ocular surgery (43). The risk of infection increases in immunocompromised patients, in those with chronic use of contact lenses, in intravenous drug users, and in those with chronic use of topical steroids.

Filamentous fungi are the most common cause of fungal keratitis, of which *Aspergillus flavus* is the most common fungal isolate reported, especially from India (6, 44). Rare forms of fungi known to cause infectious scleritis have been reported in the past. *Pseudallescheria boydii*, a saprophytic fungus commonly isolated from agricultural soil and contaminated water, has been observed to cause significant posterior sclera involvement with multiple abscesses (45). *Paecilomyces lilacinus* is an environmental mold found in soil and plants that causes human infection only in rare cases (46). Chung et al. reported on a case of *P. lilacinus* that manifested as several scleral abscesses with fibrinoid anterior chamber response that developed to invade the cornea despite topical and oral antifungals (46). Phthisis bulbi was seen despite aggressive therapy with surgical debridement and intracameral injection of amphotericin B for 4 months. *Scedosporium prolificans*, an emerging opportunistic fungus, has been reported to cause late-onset infectious scleritis following pterygium surgery with poor response to voriconazole (47).

Fungal scleritis, unlike the bacterial and viral infections, is associated with poor disease course and outcomes (42, 44). Longer latency periods and smaller necrotic areas at the initial presentation have been commonly observed, suggestive of a chronic indolent course (5). Fungal scleritis is often associated with poor visual outcomes due to delayed diagnosis, unavailability of fungicidal agents, poor penetration of the anti-fungal drugs in the avascular scleral tissue, and the ability of the organism to quiescent in the avascular tissue for prolonged periods without inciting an inflammatory response, thus delaying the clinical diagnosis. **Table 1** highlights the outcomes of fungal keratitis as reported previously.

**Table 1:** Clinical presentation, organisms, management, and outcomes of eyes with fungal scleritis (studies **≥ 10 eyes)**.

Study, year N= Total no. of eyes	No of eyes having fungal keratitis (%)	Identified risk factor (duration)	Mean duration of treatment	Species identified	Management			Outcome	Complications
					Topical	Systemic	Surgical		
Reddy et al. (10), 2015 N=42	10/42 (23.8)	Surgery: 3 (SICS-1; Injury: 3; Topical corticosteroids: 6	92 days (20 days to 1−year)	*Cladosporium Sp: 1; Dematacious sp: 3; Aspergillus Sp: 3; Paecelomyces sp: 1; Unidentified sp: 2*				Complete resolution: 9/10 LTFU: 1/10	Cataract: 2/10 Recurrence: 2/10
Pradhan et al. (13), 2013 N=12	3/12 (25%)	Surgery: 1 (pterygium); Injury: 2	NA	*Aspergillus sp: 1, Yeast* *Rhodotorula sp: 1, Unidentified: 1*	NA	NA	TPK+ Debridement: 1 FB removal+ Debridement: 1 Debridement: 1	Complete resolution: 3/3	Failed graft: 1
Sahu et al. (42), 2012 N=17	4/17 (23.5)	Injury: 1; Surgery: 1 (cataract); NA: 2	NA	*Fusarium sp: 1; Paecilomyces sp: 1* *Unidentified sp: 2*	NA	NA	NA	Complete resolution: 4	Corneal scar: 4 Cataract: 1
Jain et al. (3), 2009 N= 21	8/21 (38) Fungus alone: 5 (24%) **Mixed infection: 3 (14%)**	Surgery: 6. (3-cataract, Pterygium excision with MMC- 2, SB- 2); Injury: 1; Acid burn: 1	NA	*Aspergillus sp: 2* *Cladosporium Sp: 1* *Acremonium sp: 1* *Unidentified fungus: 1* ***Mixed infection*** *Fungus/A. fumigatus +* *Pseudomonas: 1* *Fungus/unidentified* *hyaline* *fungus + Pseudomonas: 1* *GPC/S. pneumoniae + Fusarium: 1*	Natamycin 5% eye drops	Itraconazole 100 mg BD	Buckle explantation: 1 Debridement: 3 Debridement+ Evisceration: 2 Evisceration: 2	Complete resolution: 3 Phthsis bulbi: 1 Eye eviscerated: 4	Endophthalmitis: 2 Choroidal detachment: 1 Cataract: 1

Complete resolution: Complete resolution of the symptoms, congestion, or active infiltrate.

SICS, small incision cataract surgery; NA, not available; GPC, gram positive cocci; TPK, therapeutic penetrating keratoplasty; LTFU, lost to follow up; FB, foreign body.

Mixed infection: infection caused by two or more species of organism (fungal, bacterial, viral, others) was in bold.

### 6.3 Viral scleritis

Herpes simplex virus type 1 (HSV-1) and varicella zoster virus (VZV) are most associated with infectious viral scleritis (32, 48). Herpetic scleritis is known to affect middle-aged women, with unilateral clinical findings and moderate to severe ocular pain. As per the current literature, the duration of presentation may be acute (for days) or chronic (3.2 years) (49). Lack of specific signs often result in a diagnostic delay. HSV scleritis commonly presents as unilateral diffuse anterior scleritis and may be associated with keratitis and uveitis. Presence of disproportionate loss of vision, perilimbal devascularization, adjacent corneal thinning, central corneal sparing, significant uveitis, and raised intraocular pressure, in the absence of systemic findings, should raise a suspicion of herpes scleritis. In general, herpes infections are not associated with posterior scleritis, vitreoretinal involvement, or scleromalacia perforans (32, 49).

Zoster scleritis presents as painful, persistent, circumscribed nodules with translucent areas in between and a higher risk of scleral perforation of staphyloma formation (50). A definitive diagnosis by immunohistopathological analysis of scleral biopsies, Giemsa staining of scleral tissue, electron microscopy examination, and cultures is often negative as the infection occurs secondary to viral-induced immune-mediated reaction. Clinical evidence of dendritic or stromal keratitis or positive anti-HSV or anti-VZV titers may help in establishing the diagnosis (4, 50).

Resolution of herpetic scleritis has been seen with oral acyclovir (800 mg) within 3–8 weeks, with the inflammation taking around 5–32 months to resolve (49). The diagnosis of viral scleritis is challenging, but lack of improvement with immunosuppressants and resolution only on starting anti-viral therapy, presence of corneal scars, and iris atrophic patches are clinical indicators of a viral disease.

### 6.4 Tuberculous scleritis

Mycobacterial scleritis may either result due to direct scleral invasion of the organism, mycobacterium tuberculosis (MTB), or more commonly due to immune-mediated inflammatory microangiopathy (3). Among scleritis, anterior scleritis is the most common pattern of the disease (51). These patients may often present with sclero- keratitis with uveitis. Ocular MTB infection is most often a result of hematogenous dissemination from a distant site (such as lungs) (52). The patient should have a comprehensive investigation using the Mantoux test, sputum analysis, and high-resolution chest CT scan to check for systemic involvement (main focus). For 6–12 months, anti-tubercular therapy consists of 400 mg of ethambutol taken twice daily, 300 mg of isoniazid taken once daily, 600 mg of rifampin taken once daily, and 50 mg of pyridoxine taken once daily.

Mycobacterial infections other than tuberculosis (MOTT) have a low incidence in humans but present in a disseminated form in immunocompromised patients. *M. chelonae* is among these MOTT species and has been shown to cause scleritis (53), most commonly seen secondary to vitreo-retinal surgeries. Immunocompromised immune status and distant foci of infection like spinal epidural abscess are the predisposing factors. Delayed and insidious presentation area is a hallmark of this infection. Topical amikacin eye drops (2.5%) every hour and oral rifampicin/clarithromycin or doxycycline is the preferred treatment. The *in vivo* efficacy of the drugs may be poorer than the *in vitro* sensitivity; thus, combination therapy is advocated, which may include topical ciprofloxacin/moxifloxacin/azithromycin as well. These infections often tend to recur after cessation of therapy; therefore, therapy with at least two drugs and prolonging treatment for 4 weeks to 6 months (in some cases) after the resolution of clinical signs is recommended. Topical corticosteroids are best avoided.

## 7 Diagnosis

### 7.1 Scraping

In the presence of adjoining keratitis or ulcerated scleral nodules, samples of infected areas should be taken for microbiology (3, 4). The collected material should be subjected to Gram staining, 10% potassium hydroxide–calcoflur white or trypan blue stain, and 1% acid-fast staining (in cases of suspected mycobacteria and actinomycetes infection) along with inoculation of the material on blood, chocolate, and Sabouraud’s dextrose agar. Inoculating cultures onto Löwenstein–Jensen medium and non-nutrient agar with an *Escherichia coli* overlay helps identify *Mycobacterium* and *Acanthamoeba* species, respectively, if suspected. Utmost care should be taken to avoid contamination of the collected sample with eyelashes, conjunctival tissue, and periocular skin, where the ocular commensals normally reside.

### 7.2 Tissue biopsy

**Figure 4:**
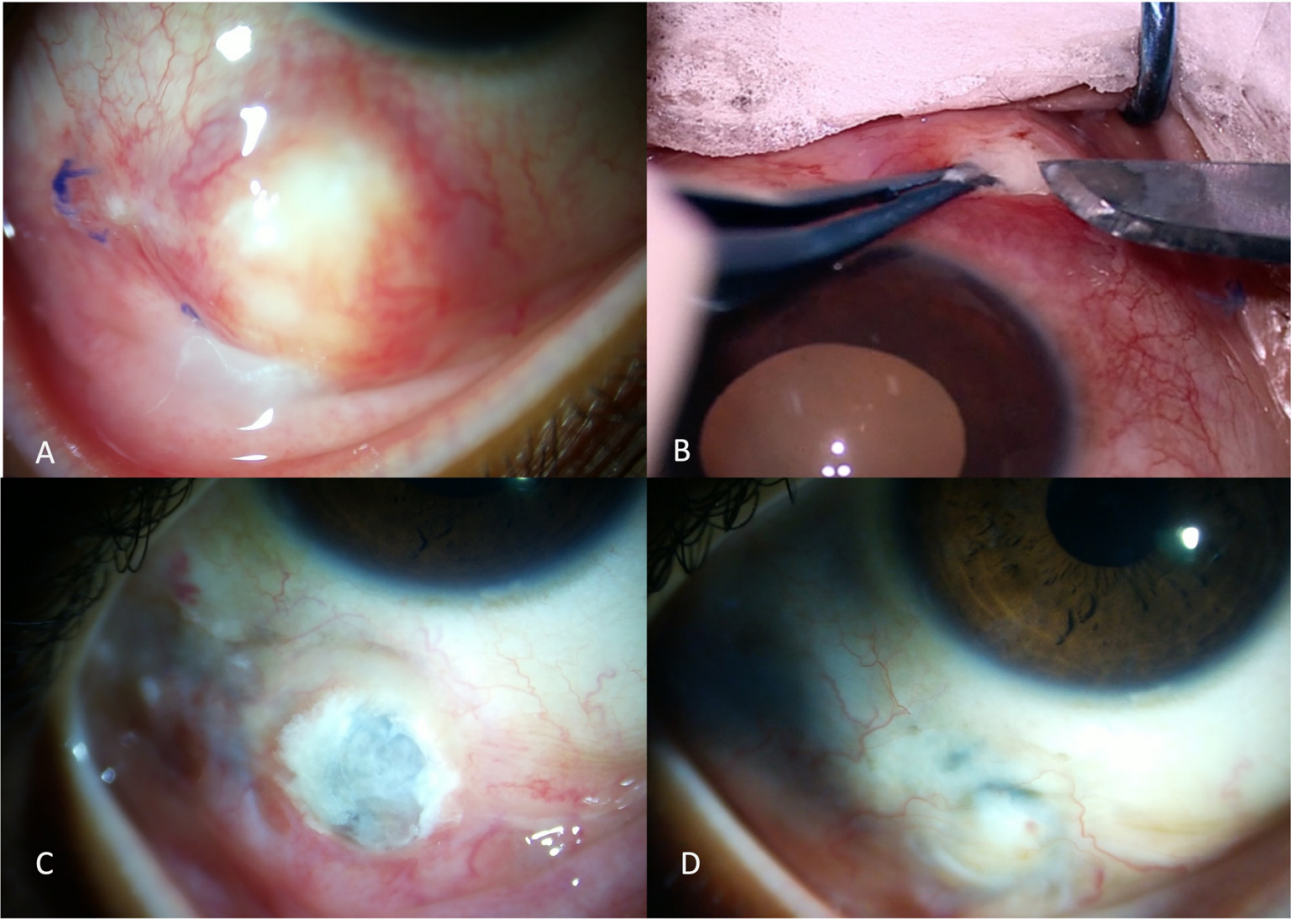
A young girl presented with a scleral nodule associated with redness and discharge for 2 months **(A)**. Surgical debridement with scleral biopsy for microbiological assessment was performed **(B)**. Scleral patch graft was performed for tectonic globe stability in view of scleral thinning **(C)**. The scleral patch graft was conjunctivalized with complete resolution of infection, two months after the intervention **(D)**.

Scleral or corneo-scleral biopsy (if there is keratitis) is often advised when microbial cultures are negative or reveal specimens with ambiguous identity, or when a poor response is seen with the initial broad-spectrum antimicrobial therapy (24). Biopsy may also be performed with the aim of debulking the infection load or when surgical intervention is anyway warranted with the aim to remove the inciting agent such as scleral buckle, foreign body, or a depot steroid injection. Tissue samples are collected directly from the depth of the lesions (**Figures 4A, B**).

### 7.3 Serological evaluation

In every case of endogenous IS, the primary focus of infection should be ascertained by performing appropriate serological testing and blood and urine cultures (26).

### 7.4 Diagnosis of posterior scleritis

The diagnosis of posterior infectious scleritis is usually made through a combination of clinical examination, imaging studies, and laboratory test. B-scan ultrasonography is invaluable for diagnosing this disease, and it reveals several signs including the thickening and edema of the posterior sclera, a scleral mass lesion suggesting an abscess, diffuse choroidal thickening, and the classic T-sign (22, 54). Ancillary tests, including enhanced-depth imaging optical coherence tomography (EDI-OCT), fundus fluorescein angiography (FFA), indocyanine green angiography (ICGA), computerized tomography (CT), and magnetic resonance imaging (MRI), have a limited role in the setting of infectious posterior scleritis but can be performed in some cases to aid in the differential diagnosis.

## 8 Management

Management of infectious scleritis (anterior and posterior) is extremely challenging, often complicated by delayed diagnosis and poor drug penetration due to avascular scleral tissue. Early recognition and appropriate treatment is important to prevent vision-threatening complications like scleral perforation, dissemination of infection, enucleation, or evisceration (5). Treatment is primarily based on the predisposing factor and the causative pathogen. Management involves a combination of medical and surgical therapy, with only 18% of patients responding to medical therapy alone and most of them requiring one or more surgical interventions (6). Preventive strategies have improved outcomes in eyes with infectious scleritis (24, 47). Post-surgical infections may be prevented by avoiding the excessive use of cautery intraoperatively and the judicious use of adjunctive therapy, especially in eyes undergoing pterygium and cataract surgery, sparing the episcleral vessels which allow better wound healing and resistance to infection (24). Bare-sclera technique of pterygium surgery has been discouraged as it increases the vulnerability of sclera to secondary infection, particularly in the setting of scleral ischemia which requires prompt intervention either in the form of conjunctival autografting or other forms of scleral reinforcement.

### 8.1 Medical management

- While awaiting microbiological reports, broad-spectrum fortified antibiotic therapy covering both Gram-positive and Gram-negative organisms should be initiated (fortified cefazolin 5% and ciprofloxacin 0.3% or tobramycin 0.3% eye drops is the most common combination therapy used), and the medication should be modified depending on the initial smear results and subsequent culture and sensitivity results (10). Various combinations of empirical treatment have been reported, with a combination of topical and systemic therapy known to provide the most favorable outcomes (3, 10, 42). Advanced cases may require intravenous antibiotics for better penetration into the sclera. A therapeutic response is expected within 24 to 48 h of initiating the antibiotics; however, minimal or no response should indicate a change in strategy and alternate therapy should be initiated (4).
- In cases with suspected bacterial scleritis, an initial apparent worsening may occur due to the bactericidal effect of the drugs and release of endotoxins from the dying bacteria, and this may need addition of topical or oral corticosteroids.
- Auto-immune scleritis must be suspected in eyes with persistently negative microbiology, despite repeated testing, and when the histopathological study of the tissue biopsy reveals an inflammatory picture. Treatment with oral corticosteroids, immunosuppressive drugs, or biologics should be considered as a last resort with or without antibiotic coverage.
- In eyes with a history of ocular trauma, particularly with organic matter, antifungal therapy like natamycin or voriconazole may be initiated, although many practitioners would prefer the use of antifungals only after the fungus etiology is confirmed. The use of empirical antifungal therapy as the primary treatment was discouraged by Murthy et al. (4) The authors argued that irrespective of history of trauma, bacterial infection is the most common etiology for IS, and hence antifungals should only be started following a documented worsening or failure on antibacterial therapy or negative smear and cultures. Moreover, response to antifungal therapy is slow and takes several weeks, during which crucial time may be lost if the actual underlying etiology is different.
- Subconjunctival antibiotics and intravenous antibiotics as an adjuvant to topical and oral antibiotics have shown positive outcomes, especially in postsurgical eyes (55).
- The technique of suprapalpebral lavage in patients with infectious keratoscleritis was initially described by Hessburg (56). Outcomes of suprapalpebral lavage therapy for the treatment of IS in eyes showing a poor response to topical fortified antibiotics was reported by Meallet in six eyes. Levofloxacin (500 mg/100 ml) and tobramycin (100 mg/L) were used for continuous irrigation of the ocular surface. The author reported several advantages of their technique apart from a continuous drug delivery system, which included continuous irrigation of the necrotic debris and the bacterial load from the ocular surface and being an extremely useful technique in patients having limited financial support and access to healthcare facility. This technique also requires minimal dependency on the healthcare staff as the tube insertion into the superior fornix required 30 min for the procedure, and the antibiotic solution bags required to be changed every 1 to 3 days for tobramycin and levofloxacin antibiotics. However, complications like postoperative hyphema, cataract formation, and systemic absorption of the antibiotics remain a major limitation.

### 8.2 Role of topical and systemic corticosteroids

g. Topical and systemic corticosteroids must be administered with extreme caution and should be monitored regularly (4, 42). Corticosteroids suppress the host immune response, causing reduced tissue destruction. Steroids should be started to suppress the inflammation only after the infection has been adequately controlled with adequate anti-infectious therapy and when suspected fungal or mixed infection is ruled out with multiple negative biopsies and histopathological tissue evaluation is suggestive of inflammatory microangiopathy.
h. The use of topical or systemic steroids in conjunction with antibacterial therapy has always remained controversial despite a few reports stating successful outcomes (10, 13, 47). When used alongside antibiotics, steroids inhibit the upregulation of host cytokines and chemokines caused by the microorganism, thus reducing the activity of the proteolytic enzymes and polymorphonuclear leucocytes causing tissue destruction. Pradhan et al. reported successful outcomes following the use of topical dexamethasone in combination with antimicrobial therapy in eyes with infectious scleritis associated with severe intraocular inflammation in 58.3% eyes (7/12) (13).
i. Successful outcomes have been reported by Huang et al. with low-dose oral corticosteroids (28). The authors claim that low-dose oral steroids eliminate the direct contact of topical steroids and reduces the risk of exacerbation or late recurrence of scleritis. Ahmad et al. reported successful outcomes in *Pseudomonas* scleritis with oral steroids. The steroids were initiated after 48 h of antibiotic therapy in microbiologically proven cases, after response was noted and fungal infection was ruled out (55). These patients (61%) achieved better visual outcomes (visual acuity >20/200) compared to those who did not (39%) after the infection resolved. Therefore, in *Pseudomonas* and other severe bacterial infections, topical and oral corticosteroids are recommended with close observation.

### 8.3 Surgical management

Pyogenic scleral infection is difficult to treat due to poor penetration of the antimicrobial drugs in the avascular necrotic sclera. Superior outcomes have been seen with combined medical and surgical intervention compared to medical therapy alone (6).

Early surgical debridement offers several benefits like providing sample for microbiology and histopathological evaluation for the early identification of the infectious agent, removing the offending material like the glaucoma drainage device or the scleral buckle and, most importantly, promoting drug penetration by reducing the infective load. Several studies have reported that on wound exploration, the area of scleral involvement is often found to be bigger than what is seen clinically (13, 26, 38, 44). Recovery is known to occur faster, and better prognosis is seen in eyes where early (within 2 to 3 weeks) surgical debridement is performed compared to delayed intervention. Multiple wound debridement, with copious irrigation of the wound with antifungals and antibiotics, helps in preventing seeding and expedites healing (6, 13, 26, 42, 44). Despite these reports, there is a significant discrepancy among practitioners about the effect of surgical debridement on the anatomical and visual outcomes in eyes with IS. Some authors have reported that early surgical debridement shortens the disease course and improves visual outcomes. With early surgical intervention, Tittler et al. demonstrated 100% globe preservation, best corrected visual acuity between 20/120 and 20/400, lesser complications, and shorter hospital stay with early surgical debridement, preferably within 2.5 days (12).

The appearance of “tunnel lesions” was initially described by Raber et al. in an eye with *Pseudomonas* keratitis, extending to involve deeper scleral structures (57). The authors believed that the presence of tunnel lesions and delayed surgical intervention contributed to the poor outcome. Similar lesions were observed by Lin et al. during early surgical debridement (26). They described a slender lesion extending only into the deeper scleral layers, with the overlying superficial sclera and conjunctival tissue being uninvolved. The authors claim that after surgical debridement, daily wound irrigation with antibiotic solution helps in the early resolution of the infection. The irrigation helps to remove the necrotic tissue, microorganism load, and proteolytic enzymes which accumulate in the deep furrows after debridement. In eyes with severe scleral thinning and underlying uveal show, a corneal or scleral patch graft may be required to reestablish tectonic globe stability (**Figures 4C, D**). In the presence of scleral thinning but with absent uveal exposure, tenon’s advancement or a multilayered amniotic membrane transplantation may be required. All of these surgical interventions must be performed when the infection is adequately controlled. Various biomaterials that have successfully restored globe integrity include autologous perichondrium grafts of the external ear cartilage, autologous fascia lata grafts (with amniotic membrane for large defects), bovine pericardium grafts, and umbilical amnion grafts, followed by amniotic membrane transplantation (5).

Poor visual outcomes despite surgical debridement were reported by Jain et al. (3) Though resolution of infection was seen in 71% eyes, useful vision (vision better than 20/200) could be attained only in 33% cases. Majority of these eyes had fungal infection.

## 9 Outcomes

In eyes with IS, outcomes following medical or surgical intervention may be defined broadly into anatomical (globe integrity) and functional (visual acuity) outcomes along with the associated complications. Though majority of the studies have demonstrated poor outcomes in eyes with IS, there is enough evidence supporting that the outcomes depend on multiple factors like early diagnosis, prompt treatment and medical or surgical intervention (47). The clinical profile and treatment outcomes of studies (including >12 eyes) are elaborated in **Table 2**.

**Table 2:** Demographics, clinical profile and management outcomes in eyes with infectious scleritis.

	Age mean (years) Male: female ratio	Risk factor	Systemic disease	Topical/ systemic steroids before presentation	Latency period between the surgery/ trauma and onset of IS	Most common clinical sign	Isolated microorganism	Treatment			Median duration for resolution of infection	Successful anatomical outcome	Complications (primary cause of poor vision)
								Topical	Systemic	Surgical			
Jain et al. (3),t 2009 21 eyes of 21 patients	6-80 years 6:1	Surgery: 19/21 (90.4%) (Cataract surgery: 6; pterygium excision: 5; scleral bucking: 3)	DM: 1/21	NA	Cataract surgery: 1month-4 years Pterygium surgery: 1month- 3 years	Ulcerative lesion (NA)	Fungus 8/21, (38%) Bacteria 10/21 (47%) Mixed infection 3/21 (14%)	21/21 (100%)	16/21 (76%)	14/21 (67%)	NA	17 (80%)	Serous retinal or choroidal detachment (5, 23.8%) Progression of cataract (5, 23.8%) Evisceration (4, 19%)
Sahu et al. (42), 2011 17 eyes of 17 patients	52.3 ± 19.75 years (range 13-75) 10:7	**Surgery (9, 52.5%)** Cataract surgery:7 Scleral buckle: 1 Trabeculectomy: 1 **Accidental Trauma: 2, %)**	DM: 1/71	Topical steroids- 15 (88%) Oral corticosteroids- 2 (11.7%)	Surgery: 1week-4 years Trauma: 3weeks -7months		Bacteria 13/17 (76%) [MC- Staphylococcus species] Fungi 4/17 (23.52%) [MC-NA]	17/17 (100%)	17/17 (100%)	13/17 (76%)	22.57 ± 19.53 weeks (range of 3–89 weeks)	17/17 (100%)	Choroidal detachment: 2; Corneal abscess:1
Hodson et al. (6), 2013, 56 eyes of 55 patients	70 years (range 5–92) NA	**Surgery: 40 (39,71%)** Pterygium surgery (mitomycin C) (radiation- 14 Cataract surgery: 9, Retinal surgery: 9, glaucoma surgery: 6, Conjunctival tumor removal- 2 **Accidental Trauma: 8 (14.5%)** **Endogenous: 8 (14.5%)**	NA	NA	Surgery: 1.9 months (0-183) Trauma: 0.2months (0-3)	Hyperemia: 55 (98%)	Bacteria 47/56 (87%) [MC: Pseudomonas spp] Fungi 7/56 (11%) [mc: Fusarium spp]	56/56 (100%)	43/56 (77%)	46/56 (82%)	46 days (range 20-71)	33/56 (58%)	Cataract: 6; Ocular hypertension/glaucoma: 3; Corneal edema: 10, Retinal complications: 6; Phthisis: 4; Perforation: 3
Pradhan et al. (13) 2013, 12 eyes of 12 patients	57± 22.86 years (range: 19-83) 4:8	**Surgery: 6 (50%)** Pterygium - 1surgery SICS- 4 Trabeculectomy-1 **Accidental trauma: 3 (25%)** **Unknown: 3 (25%)**	DM: 2/56 CLD: 1/56 Progeria: 1/56	Topical steroids 7 (58.3%)	NA	NA	Bacteria 9/12 (75%) [MC: Pseudomonas spp] Fungus: 3/12 (25%) [MC: NA]	12/12 (100%)	9/12 (75%)	5/12 (41.6%)	NA	8/12 (66.7%)	Corneal scarring following Keratitis (1), failed corneal graft (1), secondary angle closure Glaucoma and optic atrophy (1), choroidal detachment (1), Endophthalmitis (5)

Successful anatomical outcome: Complete resolution of the symptoms, congestion, or active infiltrate with medical therapy and/ or surgical intervention and preservation of the globe (even if the functional vision was not retained in the affected).

NA, not available; DM, diabetes mellitus; MC, most common; CLD, chronic liver disease; SICS, small incision cataract surgery.

Mixed infection: infection caused by two or more species of organism (fungal, bacterial, viral, others) was in bold.

Poor prognostic factors include eyes with fungal scleritis compared to bacterial infection, associated kerato-scleritis or endophthalmitis *versus* isolated scleritis, presenting visual acuity of 20/200 or worse, and medical therapy alone compared to combined medical and surgical treatment. These findings were consistently reported in several studies (3, 6, 10, 42). Fungal scleritis is associated with poor anatomical and functional outcomes due to delayed diagnosis, limited number of fungicidal agents, poor drug penetration in the avascular sclera, and the ability of the organism to remain dormant within the scleral lamellae for prolonged periods, causing progressive worsening.

Surprisingly, visual outcomes were not affected by the inciting factor (surgery, trauma, endogenous infection) or the infectious organism (6, 27, 58). Various treatment techniques such as topical medications alone, combined topical and systemic medical therapy, supra-palpebral antibiotic lavage, surgical debridement, tectonic patch graft using amniotic membrane, and corneal or scleral tissue and lamellar grafts have yielded variable outcomes. However, better prognosis is seen in eyes treated with combined topical and systemic therapy compared to topical therapy alone (10).

Repeated surgical debridement resulted in a shorter treatment course with superior overall outcomes by majority of the studies. Successful anatomical and visual outcomes were reported with a combination of early surgical debridement and medical therapy (11, 13). However, Hodson et al. reported poor functional outcomes (best corrected Snellen visual acuity less than 20/200) despite a combined medical and surgical treatment (6). In their study, 50% of the eyes did not retain functional vision. Enucleation or evisceration was required in 13% of the eyes and was more common in eyes with involvement of the adjacent structures, i.e., eyes with either associated keratitis or endophthalmitis. Therefore, the discrepancy among researchers regarding the effect of surgical debridement on anatomical and functional outcomes persists. Pradhan et al. reported better anatomical outcomes in eyes that underwent surgical debridement (13). However, unlike other studies, the authors did not find any significant difference in the visual outcomes between medically and surgically treated eyes (27, 28).

## 10 Conclusion

Infectious scleritis, though rare, is an important and impending vision-threatening cause of scleritis. With the known guarded prognosis, it is essential for eye care professionals to consider IS as a differential diagnosis of scleritis. A high level of suspicion is required when the clinical picture is not established, particularly in patients with a history of trauma or surgery, and in those with presumed immune-mediated scleritis but are refractory to immunosuppressive medications. The inciting factor should not be neglected, irrespective of the time when it occurs as the microorganism is known to remain dormant within the avascular sclera. Corneal opacities, cataract, fibrotic pupillary membranes, and repairable retina detachments are the reported sequalae following IS. The diagnosis and management are extremely challenging, requiring early identification of the causative organism with prompt institution of aggressive medical and surgical treatment for better outcomes. Recurrences are rare, but it is important to follow up regularly for a prolonged period, even after clinical resolution of the infection.
